# Lifelong football training influences miR-1303 serum expression and human breast cancer MCF-7 cells motility: a pilot study

**DOI:** 10.3389/fspor.2026.1727357

**Published:** 2026-07-14

**Authors:** Annamaria Mancini, Daniela Vitucci, Francesca Maria Orlandella, Neila Luciano, Giovanni Smaldone, Georgios Ermidis, Morten Bredsgaard Randers, Magni Mohr, Peter Krustrup, Domenico Martone, Stefania Orrù, Giuliana Salvatore, Pasqualina Buono

**Affiliations:** 1 Department of Medical, Human Movement and Well-being Sciences, University Parthenope, Naples, Italy; 2 CEINGE-Biotecnologie Avanzate “Franco Salvatore”, Napoli, Italy; 3 IRCCS SYNLAB SDN, Naples, Italy; 4 Department of Sports Science and Clinical Biomechanics, University of Southern Denmark, Odense, Denmark; 5 Centre of Health Sciences, Faculty of Health, University of Faroe Islands, Tórshavn, Faroe Islands; 6 Danish Institute for Advanced Study (DIAS), University of Southern Denmark, Odense, Denmark; 7 Department of Economics, Law, Cybersecurity and Sport Sciences, University Parthenope, Naples, Italy

**Keywords:** breast cancer, Lifelong football training, MCF-7 cell line, miR-1303, motility

## Abstract

**Introduction:**

MicroRNAs (miRNAs) are key components in the interaction between lifestyle-related risk factors and cancer development. In particular, miR-1303 expression is deregulated in breast cancer tissues, with elevated levels associated with poor prognosis. We have previously shown that regular exercise reduces the expression of this miRNA in skeletal muscle. The present pilot study aimed to evaluate the impact of lifelong football training on circulating miR-1303 expression and miR-1303 modulation on proliferation, migration, and invasion pathways in human breast cancer MCF-7 cells.

**Methods:**

Blood samples were collected from 17 veteran football players (VPG) and 17 healthy, untrained males (CG) of matching age (74.4 ± 4.3 years). Serum levels of testosterone, estradiol, IGF-1, insulin (ELISA), and miR-1303 (RT-qPCR) were measured. MCF-7 cells were treated with serum collected from VPG, CG or transfected with 25 μM miR-1303 mimic or inhibitor or miRNA mimic negative control. Cell proliferation and invasion were assessed using Wound healing assays and Transwell assay, respectively. Western blotting and RT*q*PCR were performed to evaluate the expression of key migration and invasion markers.

**Results:**

We demonstrated, for the first time, that miR-1303 expression was lower in VPG serum than in CG and paralleled the lower expression previously found in skeletal muscle. Overexpression of miR-1303 increases the proliferation and motility of MCF-7 breast cancer cells and is correlated with increased expression of β-catenin, c-Myc, and MMP2 genes and decreased expression of p21 and p27 proteins, all key markers involved in migration and invasion pathways. These events were reversed by treatment with the miR-1303 inhibitor. Interestingly, treatment of MCF-7 cells with sera from VPG, with low level of c- miR-1303, parallels the effects mediated by miR-1303 inhibition.

**Discussion:**

Our findings suggest that lifelong football training may counteract MCF-7 cell proliferation and invasion activities and that miR-1303 is one of the factors mediating the effects of long-term football training on breast cancer cells.

## 1 Introduction

MicroRNAs (miRNAs) are small (19–25 nucleotides) single-stranded non-coding RNA molecules present in the cell cytoplasm regulating several biological processes including cell death, cell proliferation and differentiation (1–3). MiRNAs are also secreted into extracellular fluids (plasma, serum, saliva), in vesicles or in combination with other proteins in a highly stable form and modulate cell-cell communications (4, 5). Since miRNAs can be isolated and quantified, they represent a non-invasive and easy-to-follow biomarker for early diagnosis and prognosis in different diseases, including some types of cancer (6).

The relationship between exercise and the expression of miRNAs is well known: in fact, different types of exercise modulate the expression of many miRNAs in different organs (7–9). In particular, exercise regulates the expression of miRNAs involved in adaptive processes such as myogenesis, glucose metabolism, mitochondrial function, and insulin sensitivity, unlike miRNAs involved in inflammatory processes and cellular stress resulted downregulated (10). Furthermore, the response of miRNAs to the exercise varies among individuals, depending on factors such as age, gender, fitness level, and health status (11). As for age, the elderly and people with non-communicable chronic diseases seem to have a more pronounced response to exercise in terms of miRNA modulation (12, 13). In addition, several studies have identified miRNAs among the main players in the crosstalk between lifestyle factors and cancer development (14–16).

The World Health Organization considers physical inactivity one of the leading risk factors fordisease development (17): in particular, it is the main risk factor for 21%–25% of breast and colon cancer cases (18), as well as a major cause of mortality and morbidity from chronic diseases such as atherosclerosis and type 2 diabetes (T2DM) (19–21).

Nowadays, it is well-known that regular exercise contributes to prevent the development of some types of cancer including breast cancer, and acts as adjuvant to improve cancer therapy response and the delay of cancer progression, in survivors (22).

Also, regular sport training engagement lifelong has been shown to affect the expression of several genes, including some miRNAs involved in the oxidative metabolism, DNA-repair and autophagy pathways leading to healthy longevity (23–25).

Among the miRNAs differently regulated by the exercise in the skeletal muscle, miR-1303 expression was found to be lower in muscle tissue from healthy Veteran Football Players (VPG) compared to untrained age matched veterans and increased in patients with non-communicable diseases like T2DM (23, 26).

Recent evidence suggests that miR-1303 is involved in different types of cancer, with higher miR-1303 expression levels in tumor tissues correlating to the onset of some type (e.g., prostate, gastric and lung cancer) (27–29) of cancer. Moreover, this miRNA could be a potential diagnostic and predictive marker of the insurgence or of the effectiveness of anti-cancer therapy in BC (30). Despite all, the molecular mechanisms of miR-1303 in BC tumorigenesis have not been fully elucidated to date, nor has the role of exercise on miR-1303 expression and on tumorigenesis features.

To address this, we first investigated the expression of circulating miR-1303 in the serum of VPG and, successively, we investigated the role of a lifelong football training and miR-1303 expression on the proliferation, migration and invasion in *in vitro* model of human BC cells (MCF-7). Here, we point-out that miR-1303 expression was lower in VPG serum compared to healthy, age matched, untrained men used a control group (CG), independently from the IGF-1, insulin and estradiol hormone concentrations. Moreover, we confirmed that miR-1303 overexpression promotes the proliferation, migration and invasion feature of human MCF-7 cells, and importantly, we demonstrated for the first time that the treatment with VPG sera counteracts cell proliferation and invasion activities in these cells.

## 2 Materials and methods

### 2.1 Sample collection

Blood samples were collected from 17 VPG (played football regularly for at least 40 years) aged 73.0 ± 4.0 years old and 17 untrained (CG) males (aged 75.7 ± 4.3 years). Recruitment for the VPG group was carried out through the Danish Football Association (DBU) and in collaboration with coaches from local football clubs near Odense, Denmark. The CG group was recruited through advertisements on social media and local newspapers in the same area. The anthropometric and clinical characteristics of the subjects participating in the study were previously reported (31). The inclusion and exclusion criteria have been thoroughly detailed previously (31).

Sera from subjects belonging to the same group (VPG or CG) were divided into 4 pools per group, each containing sera from 4–5 subjects. These pools were aliquoted and stored at −80°C prior to treatment of MCF-7 cells.

The study was conducted in line with the Declaration of Helsinki and was approved by regional ethics committee for Southern Denmark, as stated in (31). The subjects' habitual fitness level was assessed by a questionnaire. All participants signed an informed consent form.

### 2.2 Anthropometric, body composition, and hemodynamic parameters

Anthropometric and clinical assessments were performed under standardized conditions after an overnight fast and a 48-hour rest period from exercise. Body weight, height, and body composition were measured using a bioelectrical impedance analyzer (InBody 230, Seoul, Korea), and Body Mass Index (BMI) were calculated accordingly (kg/m^2^). Hemodynamic parameters were assessed after 15 min of rest in a supine position; Blood Pressure (BP) was recorded six times using an automated monitor (M7, OMRON, Vernon Hills, IL, USA) on the left arm, with the average value used for analysis. Resting Heart Rate (RHR) was defined as the lowest average value over a 1-minute period. These data, summarized in Table 1, are representative of a sub-cohort (*n* = 17 per group) to respect to Martone et al. (31).

**Table 1:** Anthropometric, body composition, and hemodynamic parameters of CG and VPG.

Variable (Units)	CG	VPG	*p -* value	ES Cohen's d	95% CI	
	(*n* = 17)	(*n* = 17)			Lower	Upper
Age (years)	75.7 ± 4.3	73.0 ± 4.0	0.065	0.66	−0.04	1.34
Height (cm)	177.1 ± 4.7	173.8 ± 5.2	0.062	0.66	−0.03	1.35
Total body mass (kg)	81.7 ± 11.6	82.0 ± 9.0	0.935	−0.03	−0.70	0.64
BMI (kg/m^2^)	26.1 ± 3.6	27.2 ± 2.9	0.331	−0.34	−1.01	0.34
Total lean body mass (kg)	54.0 ± 7.0	57.5 ± 4.7	0.095	−0.59	−1.27	0.10
Total body fat (%)	29.6 ± 3.3	25.6 ± 6.2	**0**.**023**	0.82	0.11	1.51
Resting heart rate (bpm)	64.7 ± 14.3	64.0 ± 9.9	0.724	−0.05	−0.62	0. 73
Systolic BP (mmHg)	135.3 ± 15.7	137.0 ± 13.2	0.875	−0.12	−0.79	0.55
Diastolic BP (mmHg)	78.4 ± 9.3	80.8 ± 8.0	0.437	−0.27	−0.94	0.41

Abbreviations: BMI, Body Mass Index; BP, Blood Pressure; CG, Control Group; VPG, Veteran Player Group. Data are presented as mean ± SD.

Bold: *p* < 0.05.

### 2.3 RNA extraction from sera and real-time PCR analysis

Total RNA was extracted from 200 µL of serum with the miRNeasy Serum/Plasma Advanced Kit (Qiagen, Hilden, Germany), according to the manufacturer's instructions. TRIzol reagent (Qiagen) was used to extract total RNA from sera. RNA was eluted in 16 µL of nuclease-free water. Total RNA obtained from individual VPG (*n* = 17) and CG (*n* = 17) sera were reverse-transcribed to cDNA by miRCURY LNA RT Kit (Qiagen) according to the manufacturer's instructions. Subsequently, the mature form of miRNAs was detected using the miR-1303 primers and miRCURY LNA SYBR Green PCR Kit (Qiagen). UniSp2 expression level was used as the reference gene and calculated using the 2^−ΔΔCT^ method.

### 2.4 Elisa assay

The concentrations of testosterone, estradiol, IGF-1, and insulin were determined in CG (*n* = 17) and VPG (*n* = 17) via enzyme-linked immunosorbent assay using commercially available kits: Human free Testosterone ELISA Kit, Human Estradiol ELISA Kit (Alpha Diagnostic International, San Antonio, Texas); Human IGF-1 ELISA - 1 × 96-WellStrip Microplate Kit (ELH-IGF1 Ray Biotech, USA), Simple Plex Human Insulin Cartridge (Biotechne, USA). All samples were processed according to the manufacturer's protocol and analyzed in duplicate within the same assay.

### 2.5 Cell culture

The human breast adenocarcinoma cell line MCF-7 was obtained from the Cell Culture Facility of the CEINGE, Naples. Cell-line identity was assessed through STR profiling. Cells were cultured in Dulbecco's Modified Eagle Medium (DMEM) supplemented with 10% of fetal bovine serum (FBS) and 1% of Penicillin-Streptomycin solution. Cells were maintained in a humidified atmosphere (5% CO_2_) at 37°C.

### 2.6 Transfection

MCF-7 human breast cancer cells were transiently transfected with miR-1303 mimic (*mir*Vana™ hsa-miR-1303, Ambion) or miRNA mimic Negative control (*mir*Vana™ miRNA Mimic Negative Control) or with the *mir*Vana™ miRNA inhibitor (*mir*Vana™ miRNA inhibitor hsa-miR-1303, Invitrogen). MiRNA mimic Negative Control (Ctrl) is a random sequence miRNA mimic molecule that does not produce identifiable effects on known miRNA function. All cells receiving treatment with pooled sera were simultaneously transfected with miRNA mimic Negative control. All transfections were performed using Lipofectamine RNAiMAX (Invitrogen, Thermo Fisher Scientific) according to the manufacturer's instructions.

### 2.7 Wound healing assay

MCF-7 were seeded into a 6-well plate at 2 × 10^5^ cells/ well and incubated with completed medium at 37 C and 5% of CO_2_. After 18 h, cells were subjected to starvation in DMEM supplemented with 0.5% FBS (32). When the cells grew to 80% confluence, 200 μL pipette tip was employed to scratch the MCF-7, followed by two washes with 2 mL DMEM without FBS to remove cell debris, and then transfected as specified above and incubated in DMEM supplemented with respective treatments. The pictures of the initial wound area were obtained using the microscope (Cell Discoverer 7—Zeiss). Pictures were obtained from the exact location and after 24 h and 48 h after transfection and analyzed through Image J software (33). Migration rate was calculated as % wound healing = [(Area of initial wound − Area of the wound after healing)/Area of initial wound] × 100%. Data are presented as the mean of three independent biological replicates ± S.D. For each independent experiment, technical triplicates were performed.

### 2.8 Transwell invasion assay

Cell invasion assays were executed using the 24-well plate Transwell divided into upper end and lower end by 8-*μ*m Pore Size membrane (Corning Incorporated, costar, USA). Cells were transfected (miR-1303 mimic or miRNA mimic Negative Control or with miR-1303 mimic + specific inhibitor) for 24 h and 5 × 10^4^ cells were seeded in media containing 5% serum and incubated 24 h at 37°C/5% CO2 with a 10% serum chemo attractant. Membranes were coated with Grow Dex Solution according to the manufacturer's instructions (34). Non-migrated/invaded cells were removed using a cotton swab and invaded cells were fixed in ethanol for 15 min. Fixed cells were stained with Crystal Violet (Serva) for 15 min. Cells were counted manually from 3 field/well images and analyzed through Image J software. Data are presented as the mean of three independent biological replicates ± S.D. For each independent experiment, technical triplicates were performed.

### 2.9 RNA extraction and real-time PCR analysis

Total RNA was extracted from MCF-7 cells at 24 and 48 h after transfection with the miRNeasy Kit (Qiagen, Hilden, Germany), according to the manufacturer's instructions and quantified using a NanoDropTM spectrophotometer. RNA was eluted in 40 µL of nuclease-free water. For the analysis of miR-1303 relative expression, 0.5 µg of total RNA obtained was reverse-transcribed to cDNA by miRCURY LNA RT Kit (Qiagen) according to the manufacturer's instructions. Subsequently, the mature form of miRNAs was detected using the miR-1303 primers and miRCURY LNA SYBR Green PCR Kit (Qiagen). U6 snRNA (Qiagen) expression level was used as the reference gene and calculated using the 2^−ΔΔCT^ method.

For the analysis of target genes, c-Myc and MMP2, 0.5 µg of total RNA was used in the reaction with SuperScript Reverse Transcriptase (Thermo Fisher Scientific, MA, USA). The resulting cDNA was analyzed by real-time quantitative PCR (RTqPCR) performed by Applied Biosystems™ SYBR™ Green (Thermo Fisher Scientific, MA, USA). Reaction mixtures were incubated at 95°C for 30 s, followed by two cycles at 95°C for 30 s and 95°C for 3 min and by 40 cycles at 95°C for 15 s and 60°C for 1 min. Finally, 80 cycles were run starting at 55°C and increasing the temperature by 5°C every 10 s up to 95°C. Fluorescence signals were measured during the elongation step. Data are presented as the mean of three independent biological replicates ± S.D. For each independent experiment, technical duplicates were performed.

The target mRNA expression was normalized to the levels of the polymerase (RNA) II (DNA directed) polypeptide A (Pol2A) gene using the 2^−ΔΔCT^ method. The primers used were as follows:

PolR2A Fw CAACGCACACATCCAGAACG
PolR2A Rev TCCTTGACTCCCTCCACCAC
c-Myc Fw CACAGCAAACCTCCTCACAG
c-Myc Rev GGTGCATTTTCGGTTGTTGC
MMP2 Fw GTGTGAAGTATGGGAACGCC
MMP2 Rev CCGTACTTGCCATCCTTCTC

### 2.10 Western blotting

Cells were lysed and protein blotted as previously described (23). The membranes were immunoblotted using Rabbit monoclonal antibodies against Phospho-mTOR (Ser2448), β-Catenin (6B3), p21 Waf1/Cip1, p27 Kip1 (1:1000; Cell Signaling Technology) and mouse monoclonal antibodies against glyceraldehyde- 3-phosphate dehydrogenase (GAPDH) (1:1000; Santa-Cruz Biotechnology Inc, USA.) Blots were incubated with appropriate horseradish peroxidase-conjugated secondary antibody and target proteins were visualised by ECL detection (GE Healthcare, Italy) by ChemiDoc Go Imaging System (Bio-Rad). Densitometric measurements were carried out using Quantity One software (Bio-Rad). GAPDH protein was used to estimate the total amount of loaded proteins. Each protein target was quantified as a percentage relative to the negative control on each membrane. Data are presented as the mean of three independent biological replicates ± S.D. For each independent experiment, technical duplicates/triplicates were performed.

### 2.11 *In silico* analysis

Targetscan (https://www.targetscan.org/vert_80/) (35), miRDB (https://mirdb.org) (36), STarMir (https://sfold.wadsworth.org/cgi-bin/starmirWeb.pl) (37) and miRWalk (http://mirwalk.umm.uni-heidelberg.de/) (38) online databases were interrogated on August 3, 2025 to predict the potential binding site of the miR-1303 on the 3′-UTR of the putative target genes.

KM-plotter online tool (https://kmplot.com/analysis/) (39) was accessed on August 4, 2025 to investigate the correlation between the expression of miR-1303 and the overall survival (OS) of breast cancer patients deposited in The Cancer Genome Atlas (TCGA).

### 2.12 Statistical analysis

Statistical analysis was performed using StatView software (version 5.0.1.0; SAS Institute Inc., Cary, NC, United States) and Jamovi software (Version 2.3.26.0). The Shapiro–Wilk statistical test was used to check the normality of the distributions. The Mann–Whitney *U*-test was then utilized to compare sera miR-1303 expression levels, estradiol, IGF-1, insulin concentration between CG and VPG. Non-parametric effect sizes were calculated as Rank-Biserial Correlations, with values |*r*| > 0.5 interpreted as large effects. Regarding miR-1303 expression, the observed effect size was −0.633 (large effect); based on this value and the Mann–Whitney *U*-test, a *post-hoc* power analysis confirmed that our sample size of *n* = 17 subjects per group provided a statistical power of 80% at alpha = 0.05. Student's *t*-test was used to compare sera testosterone concentration values. One-way ANOVA followed by Tukey's HSD *post hoc* test was used to compare data values in wound closure at each observed time (24 h and 48 h), Transwell invasion rate, miR-1303 levels in MCF-7, c-Myc and MMP2 at each time point, as well as for protein expression levels. Statistical tests were considered to be statistically significant when *p* < 0.05.

## 3 Results

The baseline anthropometric and clinical characteristics of the study population (*n* = 17 per group) are summarized in Table 1. These data, which include body composition and hemodynamic parameters, were adapted for the current sub-cohort from the dataset previously described by Martone et al. (31).

### 3.1 miR-1303 expression levels and testosterone, IGF-1, estradiol and insulin concentration in VPG and CG sera

**Figure 1:**
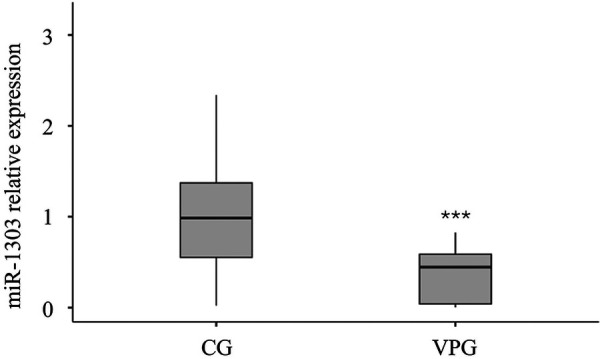
Relative miR-1303 expression in VPG and CG sera. miR-1303 relative expression in sera from VPG compared to CG by RT*q*PCR. Data represent the mean (± SD) of miR-1303 relative expression in VPG and CG sera (2^−ΔΔCT^). *P*-value was calculated by Mann–Whitney *U*-test. ***p = 0.001.

We analyzed the relative expression level of miR-1303 in the sera of VPG compared to CG (Figure 1). We found a significant down-expression of miR-1303 in the sera from VPG compared to CG (*p* = 0.001). This result parallels the lower miR-1303 expression found in the muscle of VPG compared to CG previously evidenced (23).

**Figure 2:**
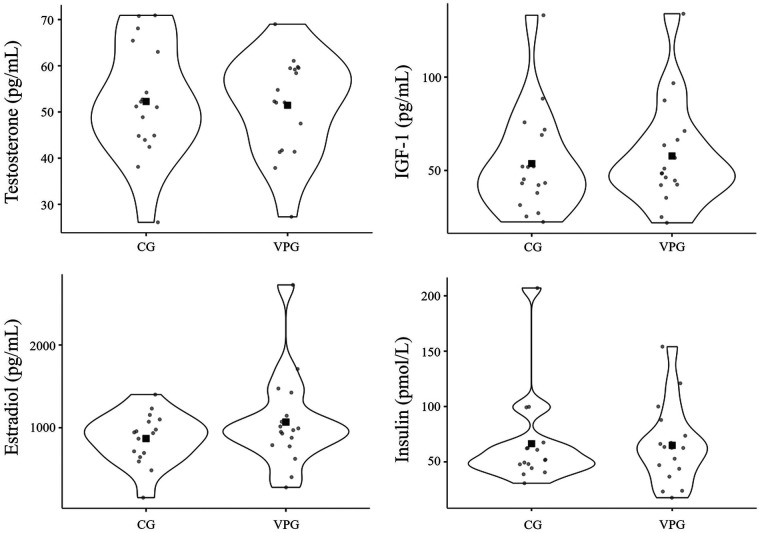
Hormones concentration in VPG and CG sera. Sera concentration of Testosterone, IGF-1, Estradiol, Insulin from VPG compared to CG. *P*-values were calculated by Student's *t*-test for Testosteron and by Mann–Whitney *U*-test for IGF-1, Estradiol and Insulin. *p* > 0.05. Black point shows median values.

To rule out potential confounding effect of different levels of some hormone involved in the cellular proliferation pathways, we measured testosterone, IGF-1, estradiol, and insulin concentration in the serum of all recruited subjects. Interestingly, no significant differences were observed in the concentration of all hormones tested between VPG and CG sera (Figure 2, *p* > 0.05).

### 3.2 Time-course of miR-1303 mimic expression in MCF-7 cells at 24 and 48h

Human breast cancer MCF-7 cells (PR+, ER+, HER2-) are derived from Luminal A breast cancer cells and represent an *in vitro* paradigm of the most representative (70%) breast cancer subtype (40). miR-1303 plays a significant role in breast cancer as demonstrated by Chen and colleagues (31). Our in-silico analysis confirmed that high levels of miR-1303 significantly correlated with poor overall survival, albeit its expression level is very low, in all breast cancer patients deposited in the TCGA by Kaplan–Meier survival curve (Supplementary Figure S1A). Kaplan–Meier survival plot also revealed that miR-1303 is unfavorable prognostic biomarker in breast cancer patients positive for ER (HR = 4.01, log-rank test *p* = 3e-07) and for PGR (HR = 4.01, log-rank test *p* = 1.3e-06) and for patients negative for HER2 (HR = 2.75, log-rank test *p* = 0.0024), since high levels of miR-1303 expression correlated with a worst survival (Supplementary Figures S1B–D). Based on these data we chosen MCF-7 cell line as cell model system for further *in vitro* experiments.

**Figure 3:**
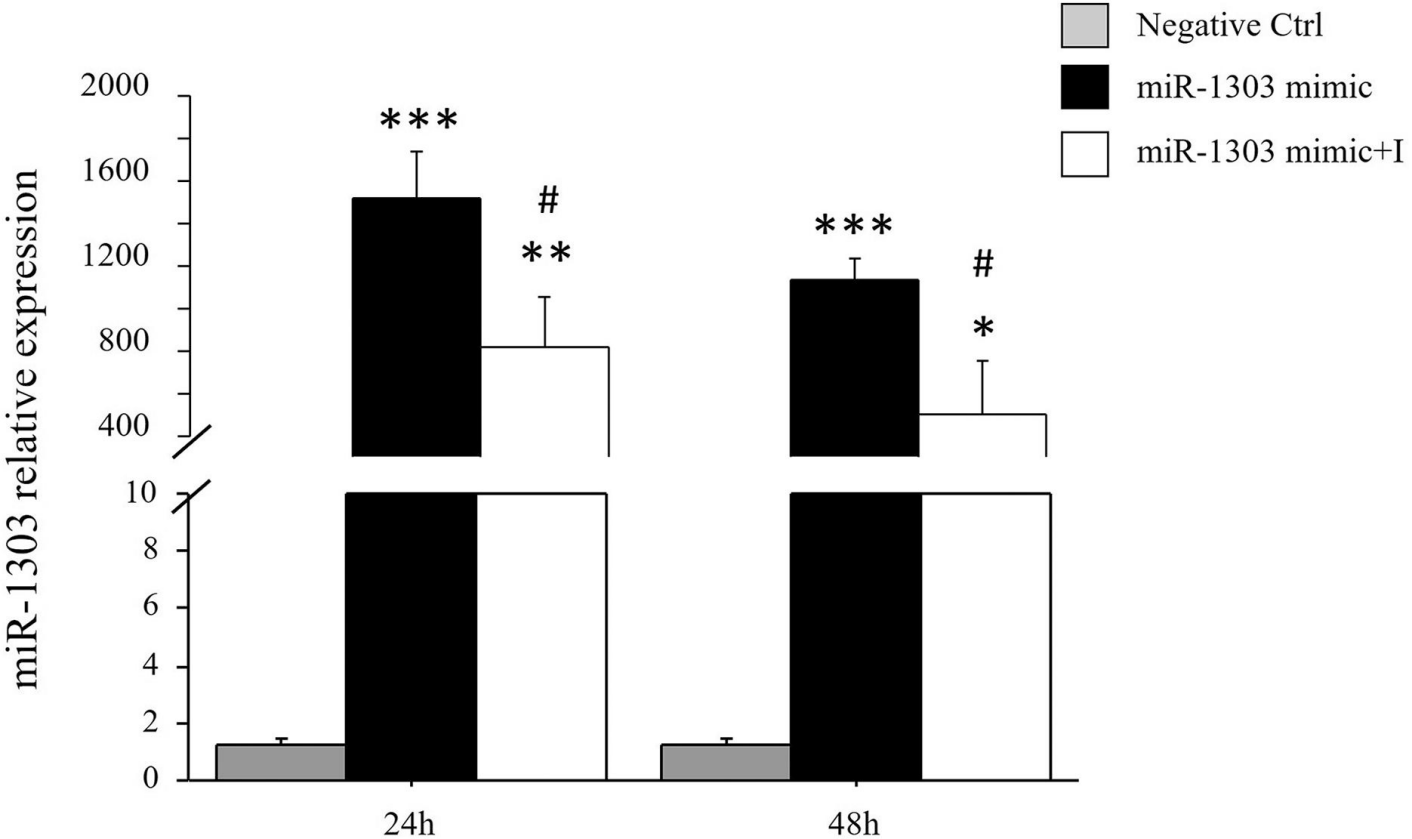
Relative miR-1303 expression in MCF-7 cells. Time-course experiment of miR-1303 mimic ectopic expression in MCF-7 cells at 24 and 48 h by RT*q*PCR analysis. Relative miR-1303 expression was determined in MCF-7 cells, transiently transfected with Negative Ctrl (gray bar), or miR-1303 mimic (black bar) alone or with miR-1303 mimic + I (white bar) at 24 and 48 h. Data represent the mean of 3 experiment (± SD of 2^−ΔΔCT^); *p*-values were calculated by one-way ANOVA for the 24 h and 48 h time points. ***p < 0.001, **p < 0.01 and *p < 0.05 vs. gray bars at 24 and 48 h, respectively; ^#^*p* < 0.05 white vs. black bars at 24 and 48 h, respectively.

We evaluated the relative miR-1303 over-expression in MCF-7 cells in a time-course experiment. The MCF-7 cells were transiently transfected with miR-1303 mimic alone or in presence of its inhibitor (miR-1303 + I) and with Negative Control; the relative expression was measured by RT*q*PCR after 24 and 48 h, respectively (Figure 3). MiR-1303 relative expression was increased of about 1 × 10^3^ fold in MCF-7 cells transfected with miR-1303 mimic alone compared to Negative control (Ctrl) at both time points (Figure 3, black bar vs. gray bar, *p* < 0.001 at 24 h and *p* = 0.001 at 48 h, respectively). Relative miR-1303 expression levels were reduced in cells co-transfected with miR-1303 specific inhibitor at 24 and 48 h compared to cells transfected with mimic alone (Figure 3; white vs. black bars; *p* < 0.05 at 24 and 48 h, respectively).

### 3.3 Effects of miR-1303 overexpression and VPG and CG sera treatment on migration and invasion of human MCF-7 cells

We performed a wound healing assay to investigate the effects of miR-1303 overexpression on the motility of MCF-7 cells.

**Figure 4:**
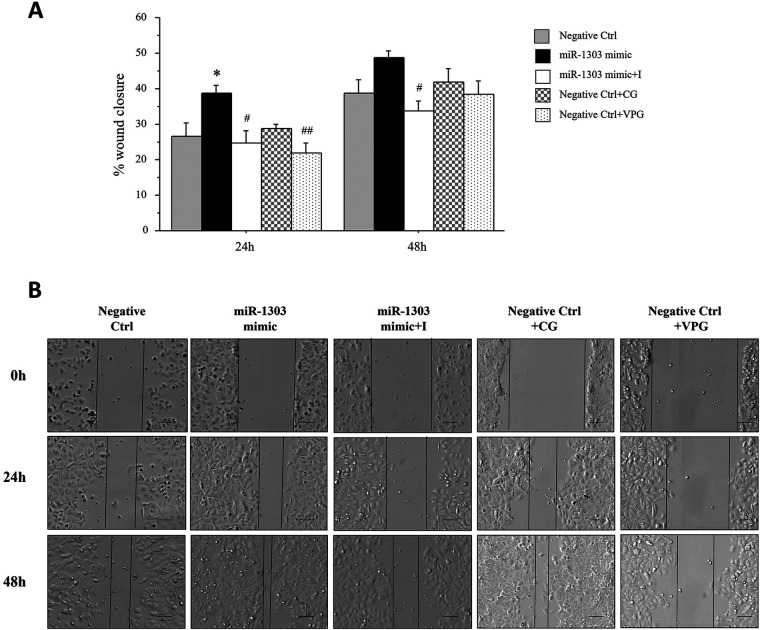
MiR-1303 overexpression and lifelong football training affect migration of human MCF-7 cells. **(A)** Wound healing closure percentage quantification of MCF-7 cells transfected with Negative Ctrl (grey bars) or miR-1303 mimic (black bars) or miR-1303 mimic + I (white bars) or treated with sera from CG (checkered bars) or VPG (dotted bars). Data are showed as the mean of three independent experiments ± S.D. *P*-values were calculated by one-way ANOVA at 24 h and 48 h time points, followed by Tukey's HSD *post hoc* test. *p < 0.05 vs. Negative Ctrl; ^#^*p* < 0.05 and ^##^*p* < 0.01 vs. miR-1303 mimic at 24 h or 48 h, respectively. **(B)** Representative bright-field microscope images of wound healing assay (scale bar = 100μm).

We transfected MCF-7 cells with Negative Ctrl, or miR-1303 mimic, or miR-1303 mimic + I or with sera from CG or VPG, respectively, and analysed the percentage of wound closure at 24 and 48 h (Figures 4A,B). miR-1303 overexpression accelerated wound closure in MCF-7 cells monolayer compared to Negative Ctrl in statistically significant manner only at 24 h, (Figure 4A, 24 h, black vs. grey bars; *p* < 0.05). The wound closure percentage was reduced in cells treated with miR-1303 mimic + I compared to the percentage observed in miR-1303 mimic transfected cells (Figure 4A, 24 h, white vs. black bars; *p* < 0.05). Further, cells cultured with VPG sera showed a reduced percentage of wound closure compared to miR-1303 mimic transfected cells and similar to cells treated with Negative Ctrl (Keywords: MCF-7 cell line; Lifelong football training; miR-1303; breast cancer; motility, 24 h, dotted vs. black bar, *p* < 0.01).

After 48 h, the percentage of wound closure was increased in all differently treated cells (Keywords: MCF-7 cell line; Lifelong football training; miR-1303; breast cancer; motility) compared to 24 h; a significant reduction in the wound healing closure percentage was evident only in cells overexpressing miR-1303 + I compared to miR-1303 mimic alone (Figure 4A, 48 h, white vs. black bar; *p* < 0.05).

**Figure 5:**
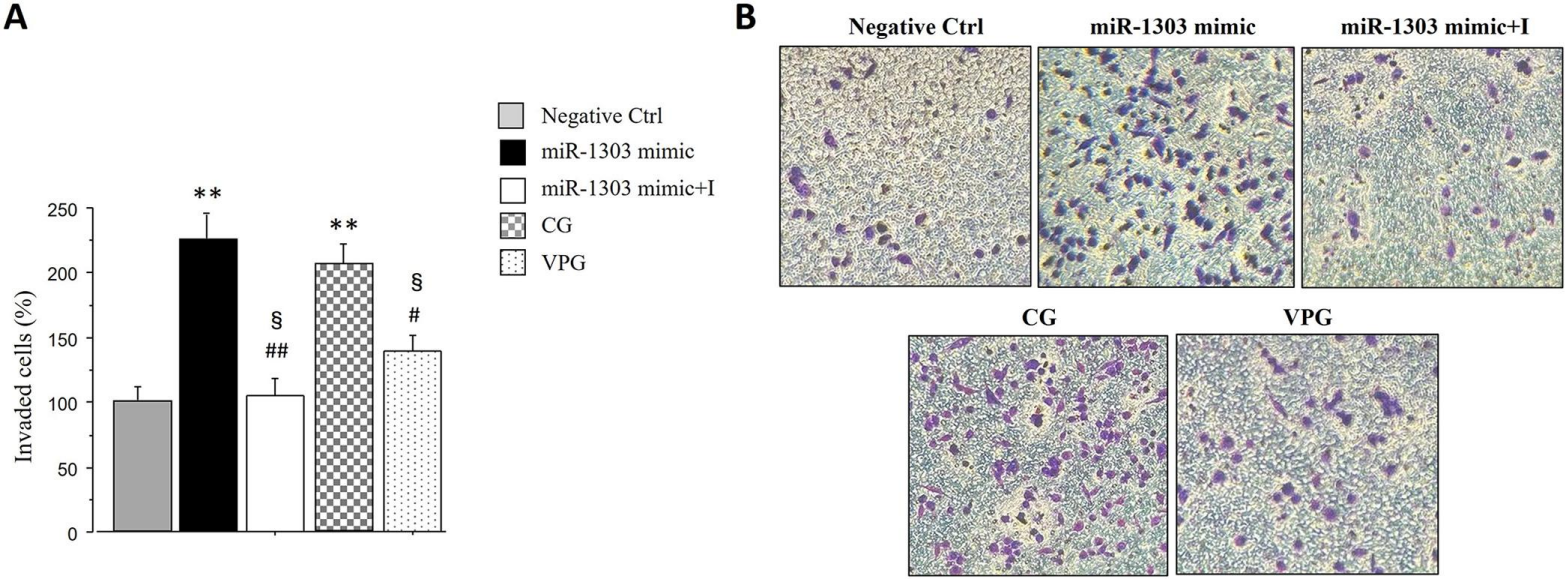
MiR-1303 overexpression and lifelong football training affect invasion of human MCF-7 cells. **(A)** Quantification analysis of the Invasion rate expressed as percentage (%) of invaded cells transfected with Negative Ctrl (grey bar) or miR-1303 mimic (black bar) or miR-1303 mimic + I (white bar) or treated with sera from CG (checkered bar) or VPG (dotted bar). Data are showed as the mean of three independent experiments ± S.D. **(B)** Representative bright-field microscope images show 10X objective magnification details. Transwell invasion assays were used to analyze MCF-7 vertical invasion. *P*-values were calculated by one-way ANOVA, followed by Tukey's HSD *post hoc* test. **p < 0.01 vs. Negative Ctrl; ^#^*p* < 0.05, ^##^*p* < 0.01 vs. miR-1303; ^§^ *p* < 0.05 vs. CG.

We also evaluated the invasion capacity of MCF-7 cells overexpressing miR-1303 alone, or miR-1303 mimic + I, or CG or VPG sera for 24 h by a Transwell invasion assay. After 24 h, the percentage of invaded cells was increased of about 2.5-fold in cells treated with miR-1303 mimic compared to Negative Ctrl (Figures 5A,B, black vs. gray bars; *p* < 0.01) and significantly reduced in miR-1303 + I treated cells compared to miR-1303 mimic (Keywords: MCF-7 cell line; Lifelong football training; miR-1303; breast cancer; motility, white vs. black bars; *p* < 0.01). Interestingly, the percentage of invaded cells was reduced in the cells treated with VPG compared to CG sera (Keywords: MCF-7 cell line; Lifelong football training; miR-1303; breast cancer; motility, dotted vs. checkered bars; *p* < 0.05).

### 3.4 Effects of miR-1303 overexpression and VPG and CG sera treatment on the expression of key proteins and genes involved in proliferation, migration and invasion pathways in MCF-7 cells

**Figure 6:**
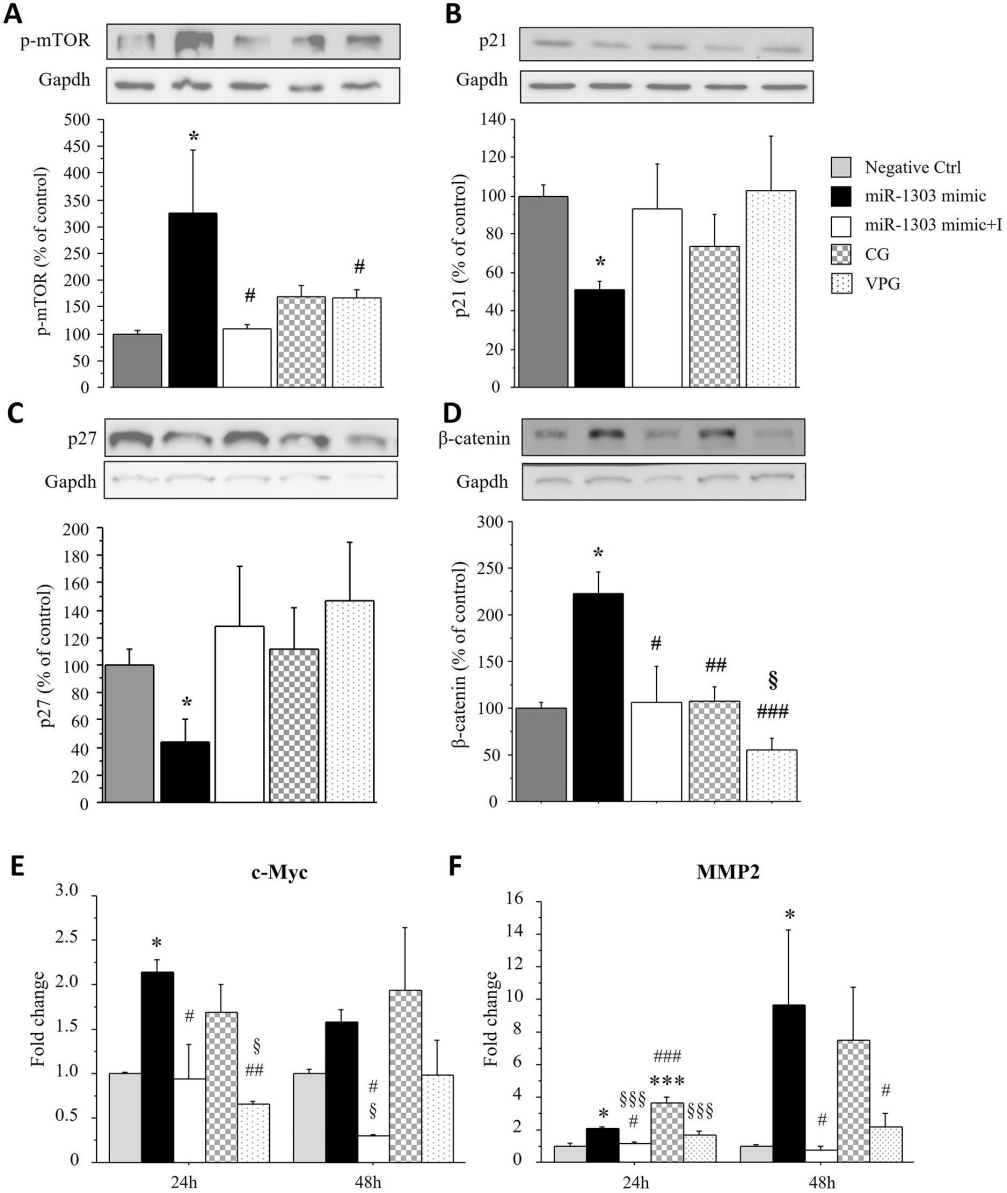
MiR-1303 overexpression and lifelong football training in MCF-7 cells parallels the expression of key proteins and genes involved in proliferation, migration and invasion pathways. **(A)** p-mTOR, **(B)** p21, **(C)** p27 and **(D)** β-catenin, protein expression analysis in MCF-7 cells transfected for 24 h with Negative Ctrl (grey bars), or miR-1303 mimic (black bars) or miR-1303 mimic + I (white bars) or treated with CG (checkered bars) or VPG (dotted bars) sera, by Western blot. Each protein target was quantified as a percentage relative to the negative control on each membrane. *P*-values were calculated by one-way ANOVA, followed by Tukey's HSD *post hoc* test. *p < 0.05 vs. Negative Ctrl; ^#^*p* < 0.05, ^##^*p* < 0.01 and ^###^ *p* < 0.001 vs. miR-1303 mimic; ^§^*p* < 0.05 vs. CG. **(E)** c-Myc and **(F)** MMP2 gene expression in MCF-7 differently treated cells by RT*q*PCR analysis. Fold change represents the mean (± SD) of genes relative expression (2^−ΔΔCT^) in Negative Ctrl, miR-1303 and miR-1303 mimic + I transfected or CG or VPG cultured MCF-7 cells for 24 h and 48 h. Data are showed as the mean of four independent experiments ± S.D. *P*-values were calculated by one-way ANOVA for the 24 h and 48 h time points, followed by Tukey's HSD *post hoc* test. *p < 0.05, ***p < 0.001 vs. Negative Ctrl; ^#^*p* < 0.05, ^##^*p* < 0.01, ^###^*p* < 0.001 vs. miR-1303 mimic; ^§^*p* < 0.05, ^§§§^*p* < 0.001 vs. CG.

To further investigate the mechanisms of miR-1303 overexpression and lifelong football training on MCF-7 proliferation and invasion activity, we analyzed the expression of key cell cycle regulatory proteins. In particular, we analyzed the expression of p-mTOR, p21 and p27 cell-cycle checkpoint proteins and β-catenin protein, in MCF-7 cells following the overexpression of miR1303. Western blot analysis suggested that, at 24 h, miR-1303 overexpression parallels p-mTOR and β-catenin protein expression increase (Figures 6A,D; *p* < 0.05) and p21/p27 check-point protein expression decrease compared to Negative Ctrl cells (Figures 6B,C; *p* < 0.05).

Furthermore, the VPG sera cultured cells showed a downregulated expression of p-mTOR (Figure 6A; *p* < 0.05) and β-catenin protein (Figure 6D; *p* < 0.001) compared to miR-1303 mimic transfected cells. Moreover, MCF-7 cells cultured with VPG sera expressed less β-catenin protein compared with CG sera treated cells (Figure 6D; *p* < 0.05).

Further, we studied c-Myc and MMP-2 gene expression, involved in the regulation of cellular proliferation and differentiation, in MCF-7 cells transfected with Negative Ctrl, miR-1303 mimic, miR-1303 mimic + I or treated with CG or VPG sera, for 24 and 48 h, respectively (Figures 6E,F) by RT qPCR. c-Myc gene expression significantly increased at 24 h in cells overexpressing miR-1303 mimic (Figure 6E, 24 h, black vs. gray bar; *p* < 0.05); this effect was reversed by miR-1303 + I treatment at 24 and 48 h (Figure 6E, white vs. black bar; *p* < 0.05, at 24 h and 48 h, respectively). Moreover, MCF-7 cells cultured with VPG sera expressed less c-Myc gene than those with CG sera (Figure 6E, dotted vs. checkered bar, 24 h; *p* < 0.05). Similarly, MMP2 gene expression was up-regulated in cells overexpressing miR-1303 (Figure 6F, 24 h and 48 h, black vs. gray bars; *p* < 0.05), this effect was reversed by miR-1303 + I treatment at 24 and 48 h (Figure 6F, white vs. black bar; *p* < 0.05). MCF-7 cells cultured with VPG sera expressed less MMP2 gene than those with CG sera (Figure 6F, dotted vs. checkered bar, 24 h; *p* < 0.001). At 48 h, the effects progressively attenuate.

We then verified if the genes investigated are directly regulated by miR-1303 by querying several online databases with different prediction algorithms. miRDB and STarMir tools predict the potential binding site of the miR-1303 on the 3′-UTR of p27 (alias name CDKN1B) (Supplementary Figure S2). This data is in agreement with the results of Chen and colleagues that demonstrated that miR-1303 suppressed the proliferation of breast cancer cells by directly targeting the 3′UTR of p27 (30).

Moreover, our search predicts a binding site of miR-1303 on the 3′-UTR of p21 (CDKN1A) (Supplementary Figures S3A,B). Nevertheless, Chen et colleagues (30) showed that the luciferase activity was not changed following the miR-1303 overexpression in MCF-7 cells.

As expected, regarding the other potential mRNA targets (mTOR, β-catenin, MMP2 and c-MYC) of miR-1303, the bioinformatics analysis on TargetScan, MiRDB, miRWalk and STarMir did not predict the miRNA-mRNA interactions.

## 4 Discussion

A large number of miRNAs have been reported to be deregulated in BC and associated with clinico-pathological features (41). At the same time, a growing body of evidence suggests that exercise may modulate the expression of various cancer-related miRNAs, including miR-21, let-7, and the miR-133 family, which are among the most frequently reported (16, 42–44). More generally, exercise-sensitive circulating miRNAs are increasingly considered part of the molecular interaction linking skeletal muscle, systemic metabolism, inflammation, and tumor biology, although the literature is still limited by small cohorts and heterogeneous training protocols (16, 45). In particular, miR-21 is a well-known onco-miR, overexpressed in many cancers and associated with cell proliferation, motility, survival, and drug resistance (42, 43). In women with BC undergoing hormone therapy, Alizadeh and colleagues reported that high-intensity interval training reduced circulating levels of miR-21, supporting the hypothesis that exercise may counteract, at least in part, a pro-tumorigenic miRNA profile (45). Similar results have been described in preclinical models, where exercise-induced reductions in serum miR-21 were associated with decreased tumor growth and angiogenesis (45–47). These observations are consistent with the broader view that exercise can attenuate tumor-promoting signaling not only through metabolic and inflammatory adaptations but also through epigenetic regulators such as circulating miRNAs (48, 49).

The let-7 family is generally considered tumor-suppressive, and reduced expression of several members of this family has been associated with more aggressive tumors (50). Several studies have reported increased circulating levels of let-7 following aerobic or high-intensity exercise in both patients and mouse models (44–46, 51). This model is intriguing because the restoration of tumor-suppressive miRNAs such as let-7 could represent one of the mechanisms through which long-term exercise contributes to creating an environment less conducive to tumor progression. At the same time, current evidence suggests caution, as the extent and direction of changes in circulating miRNAs may depend on the type of exercise, intensity, timing of sampling, and ongoing anticancer treatments (16, 48).

Members of the miR-133 family, which are involved in regulating cell motility, are reduced in the plasma of BC patients (52). However, it has been shown that resistance training and treadmill walking increase their expression in the blood of BC patients, while strength training increased circulating levels of miR-133a in prostate cancer patients, supporting a potential protective role for this family (18, 53, 54). This is also consistent with the recognized role of miR-133 as an exercise-sensitive miR, suggesting that some of the beneficial systemic effects of regular physical activity may be mediated by circulating signals of muscular origin (12).

Previously, lower expression of miR-1303 has been observed in the skeletal muscle of former professional football players (VPG) compared to controls (CG), in association with better health and longevity (23). In cancer biology, reduced expression of miR-1303 has also been associated with the inhibition of proliferation, migration, and invasion in gastric and liver cancer (28), while overexpression of miR-1303 has been linked to microsatellite instability in colorectal cancer (55). It is important to note that, in BC, previous evidence reported higher miR-1303 expression in tumor tissues, associated with a poor prognosis. From a mechanistic perspective, miR-1303 promotes proliferation, invasion, and migration by targeting p27Kip1 (30).

In the present pilot study, we demonstrated that the relative expression of miR-1303 was lower in the serum of VPGs compared to that of untrained veterans (CG), consistent with previous observations in the skeletal muscle of former football players (23). This finding reinforces the idea that lifelong football training may shape a circulating miRNA profile consistent with a healthier systemic phenotype and suggests that miR-1303 may be one of the molecular mediators linking adaptations to chronic exercise to anti-cancer signaling pathways in MCF-7 cells. Further, it is also worth considering that exercise-induced circulating miRNAs may be transported by extracellular vesicles, contributing to inter-tissue communication between skeletal muscle and tumor-related targets (48, 56).

Furthermore, we found that overexpression of the miR-1303 mimic induced proliferation and invasion in MCF-7 cells and was associated with increased expression of β-catenin, c-Myc, and MMP2, along with decreased expression of the cell cycle inhibitors p21 and p27. This molecular model is consistent with a more aggressive phenotype, as β-catenin/c-Myc signaling constitutes a key oncogenic axis and MMP2 is functionally linked to extracellular matrix remodelling and invasion. Concurrently, reduced levels of p21 and p27 are consistent with loss of cell cycle control and enhanced proliferative potential (57).

The Myc signaling pathway, which is often dysregulated in cancer, plays a central role in the suppression—mediated in part by miRNAs—of various target genes, including those encoding key proteins involved in cell cycle arrest, such as the CDK inhibitors p21 and p27 (58). Accordingly, Chen and colleagues demonstrated that overexpression of miR-1303 promotes the proliferation, invasion, and migration of BC cells and influences cell cycle progression, particularly the G2/M transition, by targeting p27Kip1, a negative regulator of the cell cycle involved in tumorigenesis (30). Our data are therefore consistent with previous evidence on BC and further support the hypothesis that overexpression of miR-1303 acts as a pro-tumorigenic miRNA in this context (30).

Taken together, our findings expand current knowledge regarding the effects of long-term football training on the expression of circulating miR-1303 and on proliferation and invasion in a cellular model of Luminal A-type human BC. Although our results have been obtained on a limited number of subjects, they support an association between long-term exercise exposure and lower levels of circulating miR-1303, although they should be interpreted with caution since circulating miRNA levels may be influenced by multiple biological variables, i.e., age, training load, sample source, and pre-analytical handling (12, 16, 48). Nevertheless, the consistency between *ex vivo* serum data, previous evidence on skeletal muscle in VPG, and the pro-oncogenic effects observed *in vitro* in MCF-7 cells suggest that miR-1303 warrants further investigation as a potential biomarker of exercise adaptation and as a potential mediator in the exercise-breast cancer axis.

## 5 Conclusions

In this study we demonstrate, for the first time, that miR-1303 expression was lower in serum from lifelong football players (VPG) compared to untrained veterans (CG). We also provided supporting evidence on the miR-1303 motility features in a subtype of BC cells, MCF-7 derived from Luminal A (ER+, PR+, HER2−) human breast cancer cells. We observed higher levels of β-catenin, c-Myc, and MMP2, coupled with reduced expression of p21 and p27, in MCF-7 cells overexpressing miR-1303.

We also confirmed that high levels of miR-1303 significantly correlated with worst overall survival, in all breast cancer patients deposited in the TCGA by Kaplan–Meier survival curve.

Interestingly, the treatment of MCF-7 cells with sera from VPG slow-down the proliferation and motility activities respect to CG sera mimicking the effects mediated by miR-1303 inhibition. Overall, these data suggest that miR-1303 is one of the players mediating the effects of lifelong football training on MCF-7 cells.

**Figure 7:**
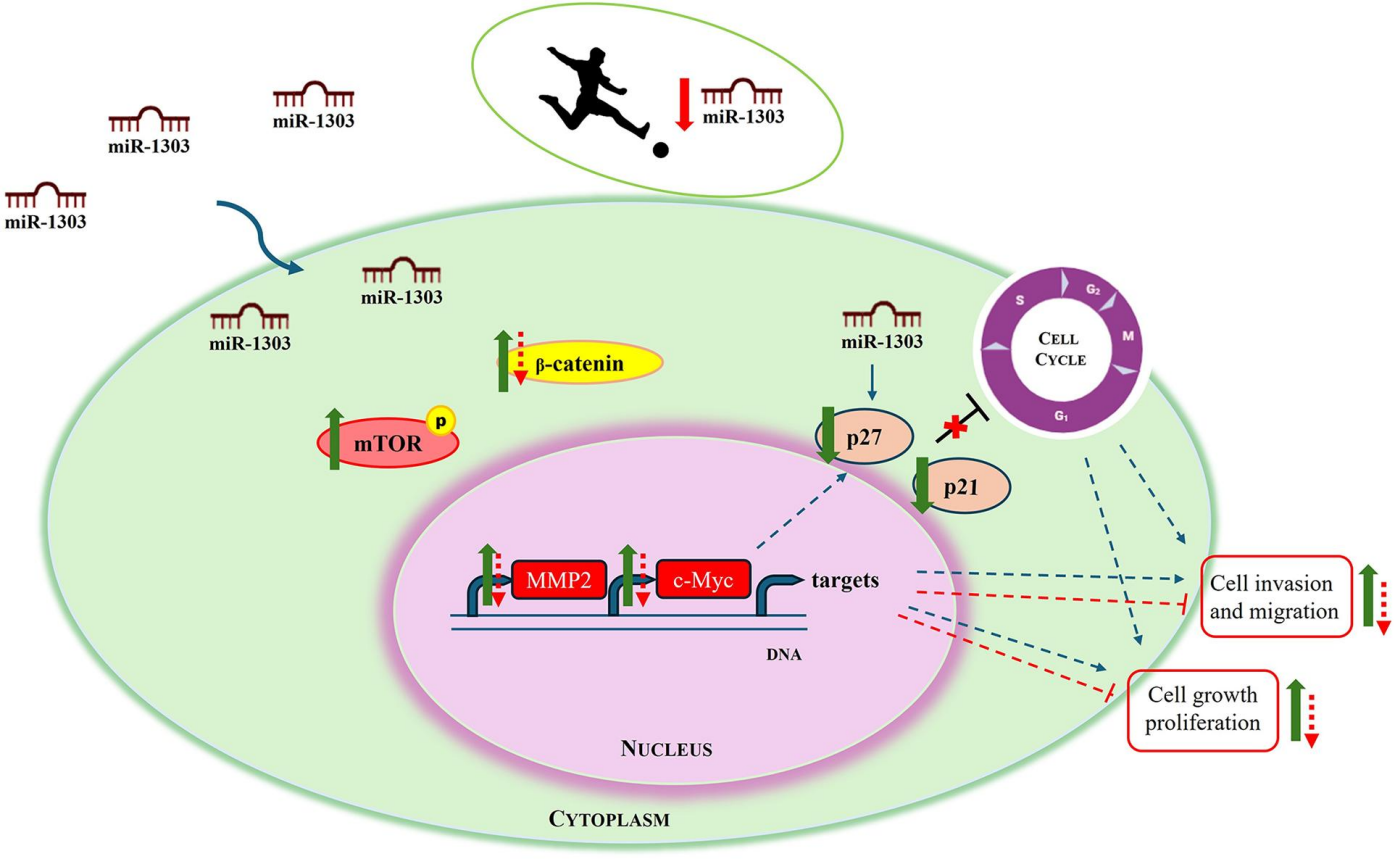
Mechanism of action overview of miR-1303 and lifelong football training on migration and invasion in MCF-7 cells. Lifelong football training downregulates miR-1303 circulating expression (red arrow), in association to reduction in β-catenin protein expression and MMP-2 and c-Myc genes transcription (dashed red arrows). MiR-1303 overexpression was associated to increase of c-Myc and MMP2 gene transcription, decreases the expression of p21 and p27 proteins and up-regulates p-mTOR and β-catenin proteins (green arrows), thereby positively regulating MCF-7 cell motility. Dashed blue arrows and red lines ending in bars indicate the modulation and inhibition, respectively, of tumorigenic events; solid lines indicate the translocation of miR-1303 into the cell.

A summary cartoon of the results obtained is reported in Figure 7. Delving into the regulatory mechanisms of this signaling pathway could aid the development of therapeutic strategies, involving the regular exercise, aimed at counteracting the proliferative and invasive activity in breast cancer cells.

## 6 Limitation of the study

Some limitations of this study should be considered. First, serum samples were pooled prior to conducting *in vitro* experiments. Although this approach allowed us to standardize experimental conditions and reduce the impact of potential outliers, it may have masked interindividual variability and prevented the performance of correlation analyses between circulating miR-1303 levels and the observed biological effects. Furthermore, although lower miR-1303 expression levels were associated with the antiproliferative cellular response, serum is a complex biological environment containing multiple bioactive components; therefore, it is not possible to establish in this study a direct causal role for miR-1303.

All the experiments were conducted on a single human BC cell line (MCF-7), that is the most representative BC (70%) subtype (Luminal A) (59). Given the well-known heterogeneity of BC, these results may not be fully generalizable to other subtypes.

Furthermore, serum samples were obtained exclusively from male participants in order to minimize hormonal confounding factors, particularly those related to estrogen. Although this choice allowed for more controlled conditions, it may limit the physiological relevance and translational applicability of the results to female populations.

Finally, the human data stems from a cross-sectional comparison between lifelong veterans and untrained individuals that does not exclude other causal inferences, i.e metabolic status and genetic background, on circulating levels of miR-1303.

Future studies addressing these aspects—including the use of a large cohort, including females, mechanistic approaches, multiple BC and prostate cancer cellular models, and longitudinal or intervention-based designs—will be performed to further validate these findings.

## Data Availability

The original contributions presented in the study are publicly available. This data can be found here: https://doi.org/10.5281/zenodo.20825885.
