# Supplementary material for: Lifelong football training influences miR-1303 serum expression and human breast cancer MCF-7 cells motility: a pilot study
Source: Front Sports Act Living. 2026 Jul 14;8:1727357. doi: 10.3389/fspor.2026.1727357 (PMC13407502; doi:10.3389/fspor.2026.1727357)
Supplement: Supplementary file 1 [file Supplementaryfile1.docx]

Supplementary Material

**Lifelong football training influence miR-1303 serum expression and human breast cancer MCF-7 cells motility.**

**Supplementary Figures**


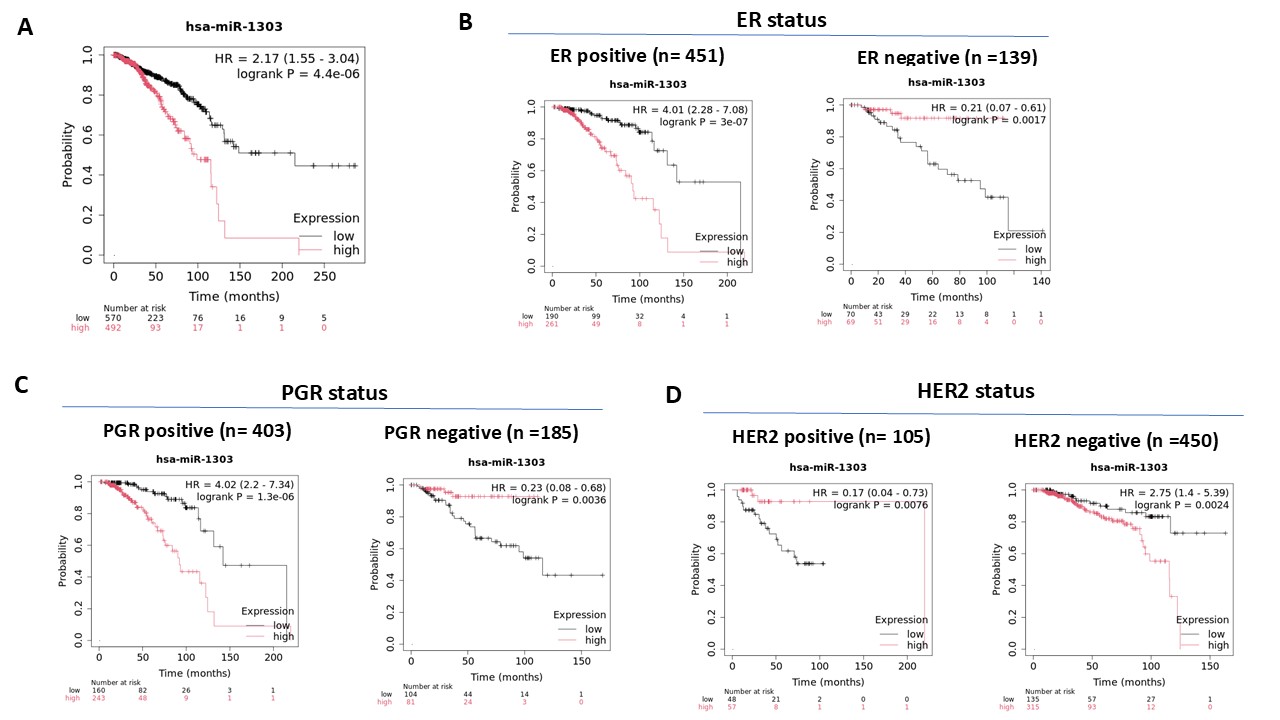


**Supplementary Figure 1***:* Kaplan–Meier analysis was obtained through an online tool (<https://kmplot.com/analysis/>), accessed on August 4, 2025. The overall survival (OS) probability in breast cancer patients deposited in The Cancer Genome Atlas (TCGA) project was calculated in the entire patient cohort (**A**) and then further analyzed by stratifying patients based on the status of Estrogen (ER) (**B**), Progesterone (PGR) (**C**) and HER2 status (**D**).


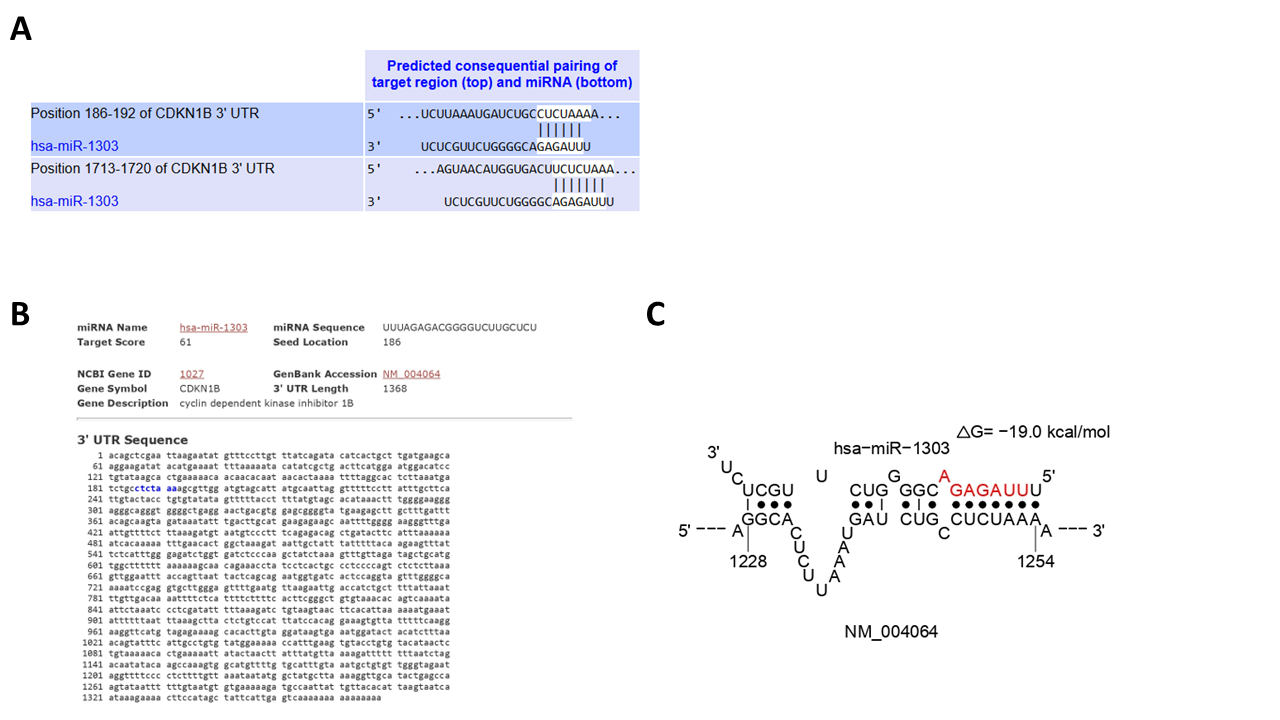


**Supplementary Figure 2**: The miR-1303 and p27 mRNA binding site predicted by TargetScan (**A**), miRDB (**B**) and the STarMir (**C**). All the online tools were accessed on August 3, 2025.


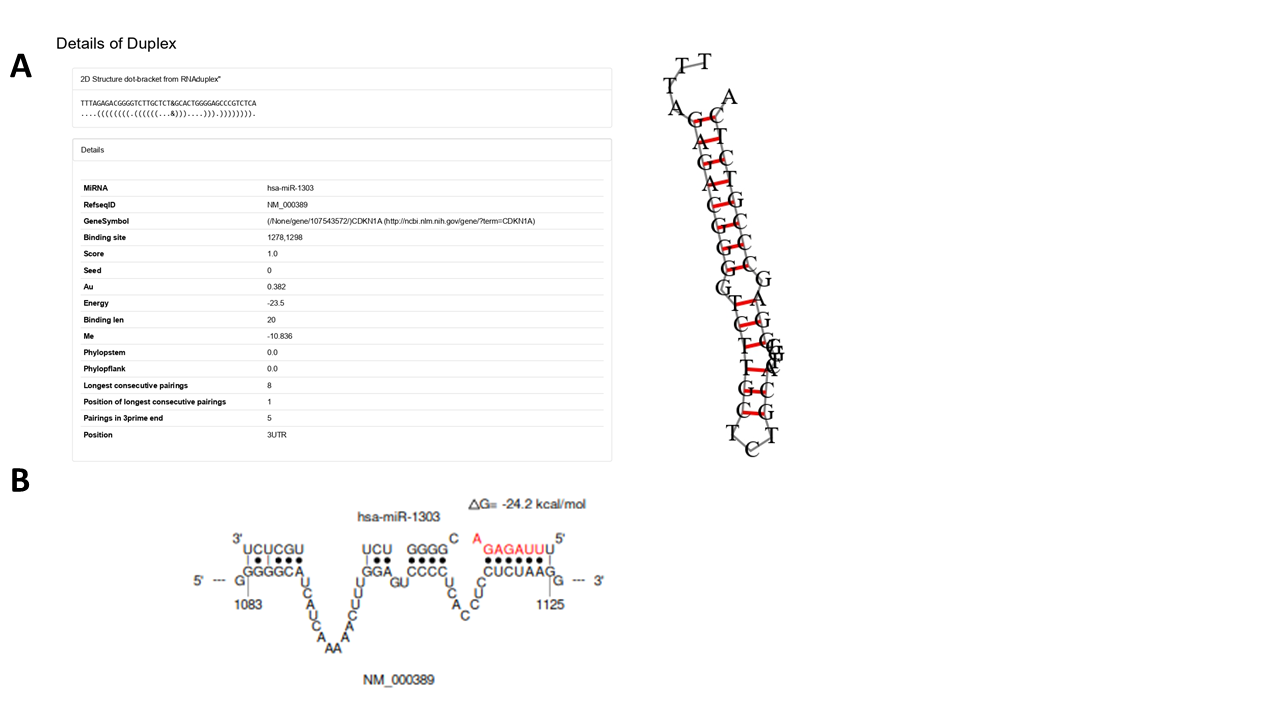


**Supplementary Figure 3:** **A.** Details (left) and 2D structure (right) of miR-1303 and p21 mRNA duplex predicted by miRWalk (accessed on August 3, 2025). **B**. The miR-1303 and p21 mRNA binding site predicted by STarMir (accessed on August 3, 2025).
